# Does the Automatic Measurement of Interleukin 6 Allow for Prediction of Complications during the First 48 h of Acute Pancreatitis?

**DOI:** 10.3390/ijms19061820

**Published:** 2018-06-20

**Authors:** Witold Kolber, Paulina Dumnicka, Małgorzata Maraj, Beata Kuśnierz-Cabala, Piotr Ceranowicz, Michał Pędziwiatr, Barbara Maziarz, Małgorzata Mazur-Laskowska, Marek Kuźniewski, Mateusz Sporek, Jerzy Walocha

**Affiliations:** 1 Department of Surgery, Complex of Health Care Centers in Wadowice, Karmelicka 5 St., 34-100 Wadowice, Poland; wkolber@wp.pl; 2 Department of Medical Diagnostics, Faculty of Pharmacy, Jagiellonian University Medical College, Medyczna 9 St., 30-688 Krakow, Poland; paulina.dumnicka@uj.edu.pl; 3 Department of Physiology, Jagiellonian University Medical College, Grzegorzecka 16 St., 31-531 Krakow, Poland; malgorzatamaraj@gmail.com; 4 Department of Diagnostics, Chair of Clinical Biochemistry, Jagiellonian University Medical College, Kopernika 15A St., 31-501 Krakow, Poland; mbkusnie@cyf-kr.edu.pl (B.K.-C.); mbmaziar@cyf-kr.edu.pl (B.M.); 5 2nd Department of Surgery, Jagiellonian University Medical College, Kopernika 21 St., 31-501 Krakow, Poland; mpedziwiatr@gmail.com; 6 Diagnostics Department of University Hospital in Krakow, Kopernika 15B St., 31-501 Krakow, Poland; mbmazur@cyf-kr.edu.pl; 7 Department of Nephrology, Jagiellonian University Medical College, Kopernika 15 St., 31-501 Krakow, Poland; marek.kuzniewski@uj.edu.pl; 8 Department of Anatomy, Jagiellonian University Medical College, Kopernika 12 St., 31-034 Krakow, Poland; msporek1983@gmail.com (M.S.); jwalocha@poczta.onet.pl (J.W.)

**Keywords:** interleukin 6, acute pancreatitis, severity, prediction of acute pancreatitis, organ failure

## Abstract

Acute pancreatitis (AP) in most patients takes a course of self-limiting local inflammation. However, up to 20% of patients develop severe AP (SAP), associated with systemic inflammation and/or pancreatic necrosis. Early prediction of SAP allows for the appropriate intensive treatment of severe cases, which reduces mortality. Serum interleukin-6 (IL-6) has been proposed as a biomarker to assist early diagnosis of SAP, however, most data come from studies utilizing IL-6 measurements with ELISA. Our aim was to verify the diagnostic usefulness of IL-6 for the prediction of SAP, organ failure, and need for intensive care in the course of AP using a fully automated assay. The study included 95 adult patients with AP of various severity (29 mild, 58 moderately-severe, 8 severe) admitted to a hospital within 24 h from the onset of symptoms. Serum IL-6 was measured using electochemiluminescence immunoassay in samples collected on admission and on the next day of hospital stay. On both days, patients with SAP presented the highest IL-6 levels. IL-6 correlated positively with other inflammatory markers (white blood cell and neutrophil counts, C-reactive protein, procalcitonin), the markers of renal injury (kidney injury molecule-1 and neutrophil gelatinase-associated lipocalin), and the markers of endothelial dysfunction (angiopoietin-2, soluble fms-like tyrosine kinase-1). IL-6 on admission significantly predicted SAP, vital organ failure, and the need for intensive care or death, with areas under the receiver operating curve between 0.75 and 0.78, not significantly different from multi-variable prognostic scores. The fully automated assay allows for fast and repeatable measurements of serum IL-6, enabling wider clinical use of this valuable biomarker.

## 1. Introduction

Acute pancreatitis (AP) is a fairly common disease, relatively mild and self-limiting in most patients. However, about 15–20% of patients develop severe acute pancreatitis (SAP) associated with serious complications in both early and late phases of the disease, causing a mortality rate of 20–30% [1,2,3].

As there is no causative treatment, clinical studies and current recommendations point towards early diagnosis of SAP, transfer to intensive care unit (ICU), implementation of supportive fluid therapy, pain relief, control of intra-abdominal pressure, and early enteral feeding as the means to improve survival in severe cases [4,5].

Difficulty in establishing universal guidelines for the management of patients with SAP arises from its dynamic progression, and even patients admitted with apparently mild symptoms may progress to SAP. The loss of local control of the inflammatory site leads to systemic activation of neutrophils and monocytes, systemic inflammatory response syndrome (SIRS), followed by organ failure [6]. In some patients, death, due to cytokine storm, may in fact occur in the first 48 h of AP (fulminant AP). Persistent organ failure is the main cause of death in the early phase of SAP, while in the late phase, the infection of pancreatic necrosis may further aggravate prognosis (critical AP) [7,8].

A wide range of diagnostic tests is used for the evaluation of AP severity in early phase, including imaging tests (primarily computed tomography with contrast, or magnetic resonance imaging), multi-variable prognostic scales (e.g., bedside index of severity in AP (BISAP)), Ranson’s scale, Glasgow scale, Acute Physiology and Chronic Health Evaluation (APACHE II), as well as single laboratory markers [9,10]. Among single biomarkers, C-reactive protein (CRP) and procalcitonin (PCT) are most widely used in daily clinical practice [11,12,13].

Since 1988, when Rinderknecht proposed a new pathophysiological concept to describe a complex network of inflammatory mediators released by activated leukocytes, clinical studies explored a wide range of inflammatory cytokines, chemokines, reactive oxygen species, adhesion molecules, and acute phase proteins as potential predictors of SAP [13,14,15,16,17]. In addition, the role of inflammatory cells and the mechanisms associated with the development of SIRS and compensatory anti-inflammatory response syndrome (CARS) in the course of SAP were extensively studied [18,19,20,21]. It is currently possible to measure the concentrations of both pro-inflammatory (tumor necrosis factor-α—TNFα, interleukins: IL-1β, IL-6, IL-8, IL-18, IL-33) and anti-inflammatory cytokines (IL-1 receptor antagonist—IL-1Ra, IL-10) [2,21,22,23,24]. Moreover, the studies by Huan et al. [25] and Manohar et al. [26] indicate that some cytokines, i.e., IL-6 and IL-22, can act as both pro- and anti-inflammatory agents [20].

Interleukin 6 is a glycoprotein with a mass of 26 kDa produced by numerous cells: monocytes, T-cells, B-cells, neutrophils, fibroblasts, and pancreatic acinar cells [2]. It has a pleiotropic effect and in acute inflammation, it restricts the synthesis of TNF-α and IL-1β with a concomitant increase in IL-1Ra synthesis and the release of the soluble TNF-α-receptor [27]. IL-6 stimulates the differentiation of antibody producing cells, macrophages, and Th17-cells [20], and induces the production of C-reactive protein from hepatocytes [1,14,15]. Interleukin 6 also acts as an anti-inflammatory mediator by stimulating IL-10 secretion [20]. Moreover, naive Th17 cells are generated in the presence of IL-6 and differentiated Th17 cells proliferate under the influence of IL-23, in turn inducting IL-23R mRNA expression [20]. In experimental AP in rats, an increase in serum IL-6 levels precede severe pancreatic edema and necrosis [28]. In humans, IL-6 is an earlier marker of severity in AP than C-reactive protein, most commonly used in clinical practice [14]. Notably, a recent study of Jain et al. [29] showed that the assessment of serum IL-6 increases the accuracy of SIRS to predict severe AP.

For a long time, measurements of serum IL-6 concentrations were not routinely available, as enzyme-linked immunosorbent assays (ELISA) did not allow for appropriately short turn-around times. In the last 20 years, significant advances in laboratory techniques enabled the development and expansion of automated assays to measure IL-6 concentrations, i.e., immunochemiluminometric assay (ECLIA), available on the Cobas Roche platform.

The authors analyzed serum IL-6 concentrations during the first 48 h from the onset of AP among patients with various degrees of AP severity. The aim of the study was to assess whether serum IL-6, measured via automatized laboratory assay, may serve as the early indicator of SAP and support a decision to urgently transfer a patient with predicted SAP to the ICU.

## 2. Results

The study included 95 patients with AP. The mean age of the studied AP patients was nearly 50 years and most patients were male (Table 1). Biliary and alcoholic etiologies of AP were almost equally common, however, one-third of patients had unknown etiology. Comorbidities were present in 44% of studied group, liver and cardiac diseases being the most prevalent. Based on 2012 Atlanta classification criteria, most patients (61%) were diagnosed with moderately severe AP (MSAP), mild AP (MAP) was diagnosed in 30% and severe AP (SAP) in 8% (Table 1). Pancreatic necrosis was observed in 13% of patients. Consequently, 75% of patients required more than one-week hospital stay. Intensive care was implemented in 7% and mortality was 4% (Table 1).

Laboratory tests, including serum concentrations of IL-6 were repeated twice: on admission (i.e., study day 1) and on the second day of hospital stay (day 2) (Table 2). No significant changes in IL-6 concentrations were observed between measurements on admission and on second day of hospital stay, regardless of AP severity (Table 2). All studied patients were admitted within 24 h from the onset of symptoms, with a subgroup of 43 patients (45%) admitted within the first 12 h. The severity of AP was comparable between patients admitted within the first 12 h and those admitted later (*p* = 0.7). We did not observe significant differences between those subgroups regarding IL-6 concentrations on admission (*p* = 0.9) and on second day of hospital stay (*p* = 0.2).

**Figure 1:**
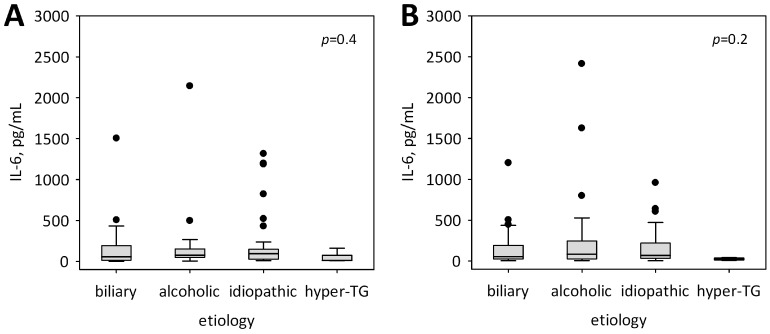
Serum concentrations of IL-6 among patients with various etiology of acute pancreatitis (AP) at admission (**A**) and on day 2 of hospital stay (**B**). Data are shown as median, interquartile range (box), non-outlier range (whiskers) and outliers (points).

Serum concentrations of IL-6 did not differ significantly between patients with various etiologies of AP, although lowest IL-6 levels were observed in AP due to hypertriglyceridemia (Figure 1).

**Figure 2:**
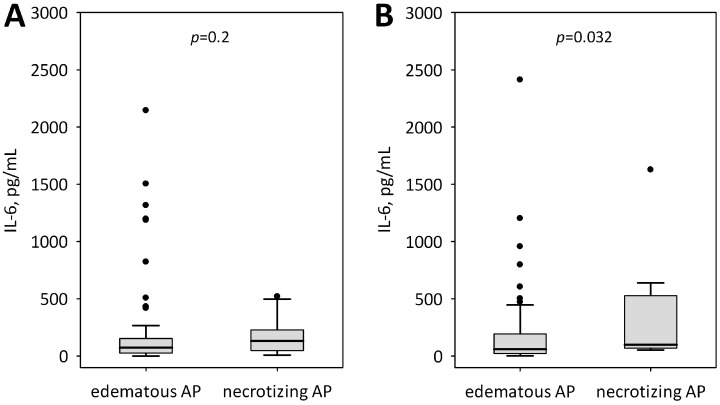
IL-6 concentrations at admission (**A**) and on day 2 of hospital stay (**B**) in edematous and necrotizing pancreatitis. Data are shown as median, interquartile range (box), non-outlier range (whiskers) and outliers (points).

Patients with SAP had highest serum IL-6 concentrations on both days of the study (Table 2). Moreover, higher IL-6 concentrations were observed among patients who subsequently developed necrotizing pancreatitis (Figure 2), however, significant difference was achieved on day 2 of hospital stay. Both on admission and on study day 2, IL-6 concentrations were positively correlated with the length of hospital stay (R = 0.27; *p* = 0.011 and R = 0.49; *p* < 0.001) as well as with Ranson’s score on study day 2 (R = 0.39; *p* < 0.001). No correlations were observed between serum IL-6 and patients’ age.

Both on admission and on day 2 of hospital stay, serum concentrations of IL-6 correlated with other inflammatory markers (albumin, C-reactive protein, procalcitonin, white blood cells, neutrophils), markers of endothelial dysfunction (sFlt-1, Ang-2), as well as the selected laboratory markers of organ dysfunction (Table 3).

Among the four components of BALI score, LDH activity exceeding 300 U/L was the one most often observed (in 83% of patients), following by high IL-6 concentrations (>300 pg/mL among 17% of patients), older age (over 65 years among 14% of patients), and urea above 8.93 mmol/L (9% of patients). Overall, 3 or 4 points in BALI score was observed in 4% of the studied group.

IL-6 concentrations measured on admission and on day 2 of hospital stay significantly predicted subsequent vital organ failure with Marshall score > 2 points and the diagnosis of SAP (Table 4). Only admission IL-6 was significant predictor of ICU transfer or death (Table 4).

**Figure 3:**
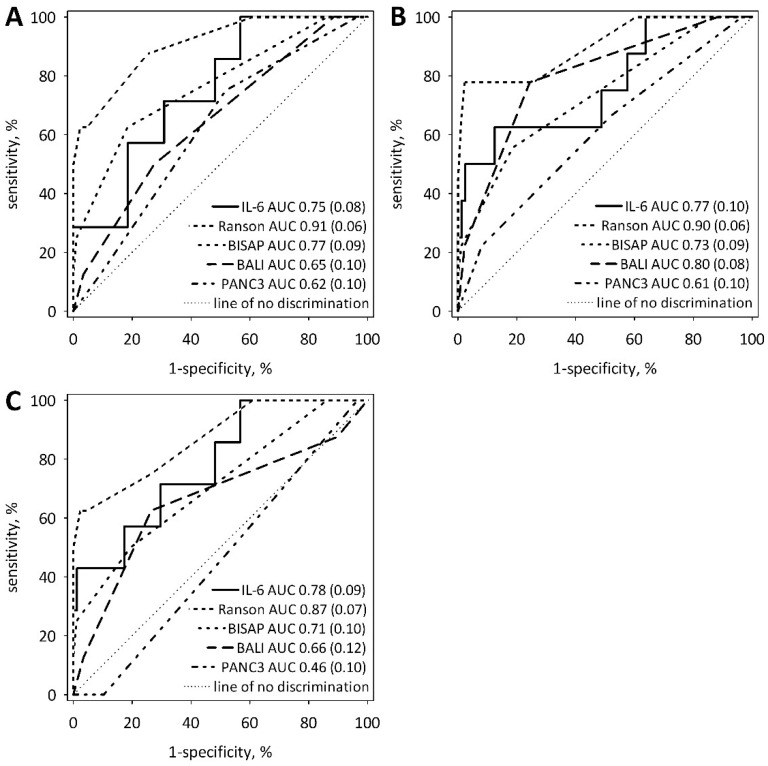
ROC curves showing diagnostic usefulness of IL-6 on admission (solid lines) in comparison to known predictive scores (Ranson’s, BISAP, BALI, PANC3) in prediction of SAP according to 2012 Atlanta classification (**A**), organ failure with 2 or more points in Marshall score (**B**), and ICU transfer or death (**C**). The values of area under the ROC curve (AUC) with standard errors (in brackets) are shown on the graphs.

Using ROC curve analysis, we compared serum IL-6 concentrations measured on admission with BALI, Ranson’s, PANC3 and BISAP scores as predictors of AP severity (Figure 3). The analysis confirmed that IL-6 on admission significantly predicted SAP diagnosis (AUC 0.753; 95% CI 0.590–0.917; *p* = 0.002), organ failure with Marshall score over 2 (AUC 0.767; 95% CI 0.578–0.956; *p* = 0.006) as well as ICU transfer or death (AUC 0.781; 95% CI 0.610–0.953; *p* = 0.001). No significant differences were observed between IL-6 and the studied multi-variable scores in prediction of SAP diagnosis according to 2012 Atlanta classification. Also, no differences between IL-6 and the studied scores were observed in prediction of cardiovascular, lung and/or renal failure (defined as Marshall score >2 points). IL-6 was significantly better than PANC3 (*p* = 0.020) and did not differ from other studied scores as a predictor of ICU transfer or death. Proposed cut-off values for IL-6 concentrations on admission are 211 pg/mL for the diagnosis of SAP (sensitivity 57%; specificity 82%); 262 pg/mL for vital organ failure (sensitivity 62%; specificity 88%); and 229 pg/mL for the prognosis of ICU transfer or death (sensitivity 57%; specificity 83%).

## 3. Discussion

The dynamic course of AP and the risk of developing systemic complications both encourage researchers to look for new early biomarkers of AP severity, re-evaluate the present ones, and verify the currently implemented prognostic scores. Important criteria for assessing the utility of predictive markers include the time needed to conduct adequate laboratory tests, but also the measurement techniques used. In the early phase of AP, life-threatening organ failure may develop during the first 48 h from the onset of symptoms. This narrows the therapeutic window and indicates the need for urgent measures to improve prognosis in predicted severe AP [30].

The majority of currently used multi-variable prognostic scales such as BISAP, Ranson’s, Glasgow, PANC3, or APACHE II employ routinely available laboratory testing methods, as well as the results of clinical monitoring and imaging. Only the BALI score proposed in 2006 by Spritzer et al. [31] utilized cytokine (IL-6) measurement as a component of prognostic assessment. Very recently, it has been shown that the addition of IL-6 improves the prediction of SAP based on SIRS criteria [29]. Nonetheless, because of the limited availability of routine IL-6 tests, BALI prognostic score has been rarely implemented. Scarcely any of the laboratories have experience in defining the cut-off points for cytokine concentrations predictive of severe AP.

Initial events in AP are associated with the premature activation of pancreatic enzymes within acinar cells. There is also evidence for nuclear factor-κB activation in acinar cells resulting in the release of cytokines and chemokines [22,32,33]. The infiltration of inflammatory cells (neutrophils, lymphocytes, and monocytes) into the pancreas promotes local injury and exaggerates inflammation [34]. The peak increase in IL-6 concentrations in systemic circulation has been shown to be an early event in experimental AP, preceding the most severe injury of the pancreas [28]. In mild AP, the inflammation remains restricted to the pancreas, while moderately severe and severe cases are associated with systemic activation of immune cells, including lymphocytes, neutrophils, and monocyte/macrophage lineage, associated with an increased expression of IL-6, IL-8, macrophage migration inhibitory factor, myeloperoxidase, neutrophil elastase, or leukotriene B4 [35]. The recent study of Jain et al. [29] points towards genetic polymorphism of the IL-6 gene (−174 G/C polymorphism) as a cause of higher serum concentrations of IL-6, among patients with more severe AP. However, the decrease in lymphocyte counts is observed in SAP simultaneously with SIRS, as well as in the increase in serum levels of anti-inflammatory cytokines, including IL-10 [35]. IL-6 as an important and early responder in the inflammatory cascade, showing also anti-inflammatory properties, seems to play an important role in the disruption of the immune system observed in SAP.

Cytokines, released in pancreatic damage, belong to low-molecular-weight proteins, physiologically present in low concentration in systemic circulation. Yet, in the course of developing AP and in response to damaging factors and inflammation triggers, their dynamic and uncontrolled increase can be observed [9]. Cytokines such as IL-1β, TNF-α, or platelet-activating factor are most often directly connected with the progression of inflammation, whereas the co-occurring activity of cytokines, i.e., IL-6, IL-8, IL-10, or free radicals in the course of AP, can affect the extent of inflammation and be associated with AP prognosis [9,15,36]. The study by Kay et al. [20] suggests that the initial inflammatory response in AP develops as naive Th17 cells are generated in response to macrophage-derived IL-6 [20], whereas the development of SAP occurs as a result of Th17 response with a greater pathogenic potential (IL-23 induced), probably due to dysfunctional autophagy or the inability of monocytes to mount an adequate IL-10 anti-inflammatory response due to anergy [20]. Moreover, Kostic et al. [21] have demonstrated that IL-6, IL-8, and IL-10 levels in the first three days of AP are accurate markers of necrosis and of a potentially lethal outcome. Observations made by Kostic et al. [21] of significantly higher IL-6 levels in patients with necrotizing AP, remain consistent with the results presented in this article.

In our study, patients who subsequently developed necrotizing AP presented higher serum IL-6, however, the difference from those with edematous AP became significant on day 2 of the study. Nevertheless, IL-6 on admission significantly predicted SAP diagnosis, organ failure with a Marshall score ≥ 2, as well as ICU transfer or death. IL-6 concentrations in patients with SAP were significantly higher compared to patients with MAP and MSAP, although the difference was more significant on day 2 of hospital stay. No significant changes were observed between admission and day 2 serum IL-6 levels. IL-6 concentrations on each of the two days were positively correlated with the length of hospital stay as well as Ranson’s score. Moreover, interesting correlations were observed between IL-6 and the early markers of acute kidney injury (KIM-1 and L-FABP) as well as the markers of endothelial dysfunction (Ang-2 and sFlt-1) [17,37,38].

In literature, a number of valuable studies justify the need for IL-6 assessment in the early prediction of AP severity [1,2,9,11,21,30], however, in light of contradictory information regarding the analytical aspect of IL-6 measurements and prognostic utility of different assays in the first 48 h of AP, implementation of this diagnostic procedure requires re-evaluation.

Serum IL-6 measurements are still rarely conducted on routine basis despite the availability of automatized IL-6 assay. In the present study, we evaluated the diagnostic usefulness of IL-6 test using a fully automated analytical platform operating on routine daily basis. The performance of the analytical procedure was controlled according to standard laboratory quality control practice and provided repeatable, reproducible results, in compliance with the requirements of Good Clinical and Laboratory Practice. We believe that for the evaluation of the diagnostic usefulness of IL-6, the measurements should be fully automated, standardized, and conducted in real time. The results of IL-6 measurements obtained by means of ELISA technique are carried out in series, after the collection of entire set of specimens and although they give valuable information on the dynamics of IL-6 changes in the course of AP, they do not permit the clinician to accumulate experience based on day-to-day practice. 

In order to predict the unfavorable outcome in the course of AP, we estimated the best cut-off points for IL-6. The adopted values are slightly lower in comparison to those proposed in BALI score (IL-6 > 300 pg/mL) [31]. In our study, serum IL-6 > 211 pg/mL on admission predicts SAP, whereas IL-6 > 262 pg/mL indicates the risk of vital organ failure. The optimal cut-off point for predicting ICU transfer or death is >229 pg/mL. The diagnostic accuracy of IL-6 measured on admission was not significantly different as compared to most multi-variable prognostic scores, including Ranson’s score (that requires the results of multiple laboratory tests done both on admission and on the following day).

Our study is not without limitations. Firstly, the percentage of patients with MAP is small as compared to most reports, including the recent epidemiological data from Eastern Europe published by Párniczky et al. [39]. In this analysis of 600 patients with AP, 61.2% were diagnosed with MAP according to 2012 Atlanta classification, while in our study only 30.5% of patients had MAP. This is related to the practice of the hospital where our patients were recruited. In many Polish hospitals, patients with predicted mild AP are admitted to non-surgical wards (gastroenterology or internal medicine). Our study included patients admitted to surgical ward, i.e., those who presented with more severe symptoms or SIRS, developed early local complications, or had comorbidities posing a higher risk for MSAP/SAP. For this reason, our cohort is “enriched” in MSAP patients. The proportion of patients with SAP (8.5%) is comparable to the data of Párniczky et al. (8.8%) [39]. Secondly, the percentage of patients who received antibiotic treatment is high, taking into account that current guidelines (including Polish ones) do not support preventive antibiotics [4,40]. This is in part associated with the severity issue. Antibiotics were introduced empirically when patients, retarded clinically in the course of AP, developed sustained fever, or when inflammatory markers including procalcitonin significantly increased. In most patients, antibiotics were not administered during the two initial days of hospital stay, so the concentrations of studied laboratory markers were not significantly affected by this treatment. Finally, the percentage of patients diagnosed as idiopathic AP is relatively high. A part of these patients was referred for further diagnostics to the tertiary center, but we were unable to collect the follow-up data.

Still, our results indicate that automatically measured serum IL-6 is a useful biomarker in the prediction of the unfavorable course of AP in a setting of secondary care hospital, i.e., a place where most patients with AP are initially admitted. A larger multicenter study is needed to allow for a generalization of the results and wider clinical uptake of IL-6 measurements.

## 4. Materials and Methods

This was a prospective, observational study carried out in the setting of a secondary care hospital. The study was conducted in Surgery Unit, Complex of Health Care Centers in Wadowice, Poland from January 2014 until December 2015. Patients were recruited within 24 h of the time of hospital admission. The study included adult patients diagnosed with AP. AP diagnosis was based on the revised 2012 Atlanta classification system [19]. Patients whose symptoms lasted for longer than 24 h before admission were excluded from the study. Moreover, patients with chronic pancreatitis, disseminated neoplastic disease, pancreatic cancer, and chronic liver disease were excluded. Only the patients who gave their written informed consent to participate in the study were included. The study protocol was approved by the Bioethics Committee of the Beskidy Medical Chamber (approval number 70/2014/B issued on 6 February 2014).

The severity of AP was defined according to the revised Atlanta classification system [10,19]. Patients who did not present any local/systemic complications nor organ failure were assigned to the mild acute pancreatitis (MAP) group. Those with local/systemic complications and transient organ failure (lasting less than 48 h) were assigned to the moderately severe acute pancreatitis (MSAP) group. The group with severe acute pancreatitis (SAP) comprised patients with persistent organ failure (which did not resolve within 48 h) in the early or late phase of the disease [19]. Vital organ (cardiovascular, respiratory, renal) failure was defined according to Marshall score [19]. Necrotizing AP was diagnosed in patients who developed (peri-) pancreatic necrosis at any time during hospitalization while those with no evidence of necrosis were diagnosed with edematous AP.

On admission, demographic data were recorded, patient’s history were collected, and a detailed physical examination was done. On admission (study day 1) and on the day following admission (study day 2), all patients underwent laboratory testing, including a complete blood count, biochemical tests (i.e., albumin, total protein, total calcium, glucose, urea, creatinine, bilirubin, aminotranspherases, C-reactive protein, lactate dehydrogenase (LDH)), coagulation system and urine tests. Routine tests were conducted in a Diagnostic Laboratory in Wadowice. The data was used to compute values of selected prognostic scales (BISAP, Ranson’s, PANC3, and BALI scores). The BALI score includes the following: blood urea nitrogen (BUN) ≥ 25 mg/dL (equal to urea ≥ 8.93 mmol/L), age ≥ 65 years; serum LDH ≥ 300 U/L; and serum IL-6 ≥ 300 pg/mL) [31]. PANC3 score comprises the following: hematocrit > 44%; pleural effusion on chest X-ray; and BMI > 30 kg/m^2^ [3]. BISAP includes five components: BUN ≥ 25 mg/dL (or urea ≥ 8.93 mmol/L); impaired mental status; presence of SIRS; age > 60 years; and evidence of pleural effusion [41]. Ranson’s score was computed using appropriate data from days 1 and 2 of hospital stay [10].

The serum and urine samples used in the routine laboratory testing also served for additional tests. The samples were aliquoted, frozen, and stored for a period of no longer than 6 months. The measurements of serum angiopoietin-2 (Ang-2), urine kidney injury molecule-1 (KIM-1), and urine liver-fatty acids binding protein (L-FABP) were conducted in the Department of Diagnostics, Chair of Clinical Biochemistry, Jagiellonian University Medical College, Krakow, Poland using ELISA reagent kits that include the following: Quantikine Angiopoietin 2 ELISA (R&D Systems, Minneapolis, MN, USA); Human Quantikine TIM-1/KIM-1/HAVCR Immunoassay from (R&D System, Minneapolis, MN, USA); and L-FABP (CMIC Holdings Co., Tokyo, Japan). For Ang-2, the minimum detectable dose was 8.29 pg/mL, and the mean serum concentration in healthy volunteers was 2494 pg/mL (range 1065–8907). For KIM-1 assay, the minimum detectable dose was 0.009 ng/mL. The normal range provided by the manufacturer was from 0.156 to 5.33 ng/mL. For L-FABP, the sensitivity of the assay was 3 ng/mL.

The concentrations of IL-6 and soluble fms-like tyrosine kinase-1 (sFlt-1) were measured by ECLIA on Cobas 8000 analyzer (Roche Diagnostics, Mannheim, Germany) in the Diagnostics Department of University Hospital in Krakow.

### Statistical Analysis

Categorical data were reported as numbers (percentage). Quantitative data were reported as median (lower-upper quartile) for non-normally distributed variables and mean ± standard deviation for normally distributed variables (as assessed with Shapiro-Wilk’s test). Kruskal-Wallis ANOVA or one-way ANOVA were used to study differences between groups based on AP severity. Spearman’s rank correlation coefficients were used to study correlations of IL-6 (considering non-normal distribution of IL-6 concentrations). Logistic regression was used to study the association of IL-6 concentrations with outcome variables (SAP, organ failure, ICU transfer or death). ROC curves were used to assess diagnostic utility of IL-6 in comparison with multi-variable scores. The tests were two-tailed, and the results were considered significant at p-value below 0.05. The calculations were made with the use of Statistica 12 software (StatSoft, Tulsa, OK, USA).

## 5. Conclusions

Serum interleukin-6 (IL-6) has been proposed as a biomarker to assist in the early diagnosis of SAP, however, most data come from studies utilizing IL-6 measurements with ELISA. Our study confirms the diagnostic usefulness of fully automatized IL-6 measurements to predict the development of SAP, vital organ failure, and the need for intensive care or death from AP. The fully automated assay allows for fast and repeatable measurements of serum IL-6, enabling the clinicians to incorporate the result in routine diagnostic reasoning.

## Figures and Tables

**Table 1:** Clinical characteristics of the study group of 95 patients with AP.

Characteristics	Observed Values
Mean age ± SD, years	48 ± 16.5
Males, *n* (%)	65 (68)
Severity of AP	
MAP, *n* (%)	29 (30.5)
MSAP, *n* (%)	58 (61)
SAP, *n* (%)	8 (8.5)
Etiology	
Biliary, *n* (%)	27 (28)
Alcoholic, *n* (%)	29 (31)
Alcohol plus high-fat diet, *n* (%)	12 (13)
Idiopathic, *n* (%)	20 (21)
Hipertriglicerydemia, *n* (%)	5 (5)
Other, *n* (%)	2 (2)
Median duration of hospital stay (Q1–Q3), days	12 (8–15)
SIRS in first 24 h, *n* (%)	74 (78)
Necrosis, *n* (%)	12 (13)
Early/late mortality, *n* (%)	1 (1)/3 (3)
BISAP score ≥ 3 in first 24 h; *n* (%)	21 (22)
Ranson score ≥ 3 in first 48 h; *n* (%)	29 (30.5)
BALI scale during first 48 h ≥ 3 points; *n* (%)	4 (4)
PANC3 score positive in first 24 h, *n* (%)	9 (9)
Harmless AP (according to HAPS), *n* (%)	46 (48)
Pre-existing comorbidities, *n* (%)	42 (44)
Cardiac diseases, *n* (%)	31 (33)
Liver disease, *n* (%)	32 (34)
Diabetes, *n* (%)	8 (8)
Dyslipidemia, *n* (%)	3 (3)
Chronic kidney disease, *n* (%)	2 (2)
Other comorbidities, *n* (%)	4 (4)
Antibiotic treatment, *n* (%)	87 (91.5)
Therapeutic ERCP, *n* (%)	5 (5)
Surgery, *n* (%)	8 (8)
Enteral feeding via nasojejunal tube, *n* (%)	10 (10.5)
Parenteral feeding, *n* (%)	3 (3)
Transfer to intensive care unit, *n* (%)	7 (7)

AP—acute pancreatitis; *n*—number of patients; MAP—mild acute pancreatitis; MSAP—moderately severe acute pancreatitis; SAP—severe acute pancreatitis; SD—standard deviation; Q1—lower quartile; Q3—upper quartile; SIRS—systemic inflammatory response syndrome; BISAP—bedside index of severity in AP; HAPS—harmless acute pancreatitis score; ERCP—endoscopic retrograde cholangiopancreatography.

**Table 2:** The results of laboratory tests on admission (day 1) and on day 2 of hospital stay according to severity of AP.

Variables	Study Day	Median (Q1-Q3)			
		MAP (*n* = 29)	MSAP (*n* = 58)	SAP (*n* = 8)	*p*
Interleukin 6, pg/mL	1	64.7 (14.8–95.7)	78.9 (27.8–163.0)	210.7 (73.1–2145.0)	0.037 ^a^
	2	38.7 (9.2–103.3)	66.9 (33.6–219.5)	280.2 (98.9–528.2)	0.004 ^a,b^
sFlt-1, pg/mL	1	129 (119–169)	140 (112–154)	191 (155–536)	0.1
	2	113 (108–145)	129 (101–161)	156 (146–209)	0.2
Ang-2, ng/mL	1	2.96 (2.03–3.64)	3.19 (2.39–3.72)	8.68 (5.11–18.8)	0.1
	2	3.47 (2.96–6.69)	3.09 (2.55–4.05)	9.02 (5.34–19.8)	0.041 ^b^
CRP, mg/L	1	22.7 (5.30–132.4)	25.4 (11.9–174.7)	129.6 (17.4–316.7)	0.4
	2	164.7 (40.4–313.6)	268.6 (97.0–371.3)	384.8 (334.7–415.2)	0.015 ^a^
Albumin, g/L	1	41.0 (31.0–44.0)	35.0 (33.0–37.0)	37.0 (35.0–39.0)	0.6
	2	36.0 (30.0–38.0)	32.0 (29.0–35.0)	24.0 (20.0–34.0)	0.033 ^a^
PCT, ng/mL	1	0.10 (0.05–0.55)	0.17 (0.10–0.36)	0.61 (0.14–1.03)	0.1
	2	0.22 (0.05–0.59)	0.46 (0.14–1.43)	1.76 (0.84–5.29)	0.009 ^a^
Total protein, g/L	1	78.0 (65.0–80.0)	65.0 (61.0–74.0)	76.5 (76.0–77.0)	0.017 ^c^
	2	66.7 (64.0–69.0)	60.0 (53.0–64.0)	62.0 (59.0–66.0)	0.012 ^c^
Hematocrit, %	1	42.4 (38.1–45.7)	43.8 (40.6–46.6)	44.5 (42.3–49.4)	0.4
	2	37.6 (36.5–39.8)	38.1 (36.3–41.5)	41.7 (37.1–45.7)	0.3
WBC, ×10^3^/µL	1	12.4 (9.5–15.2)	13.1 (10.4–16.2)	17.1 (10.3–23.3)	0.5
	2	8.6 (6.6–11.1)	10.4 (7.5–13.4)	13.7 (8.19–17.0)	0.054
NEU, ×10^3^/µL	1	11.8 (6.9–14.9)	10.5 (7.5–14.0)	9.2 (4.7–30.2)	0.9
	2	5.9 (4.3–8.1)	8.1 (5.7–11.3)	10.6 (5.6–13.1)	0.056
Bilirubin, µmol/L	1	23.4 (13.5–38.5)	27.2 (13.8–53.3)	29.1 (16.2–36.9)	0.8
	2	18.3 (13.2–27.0)	18.9 (13.7–28.2)	40.3 (39.6–43.3)	0.1
LDH, U/L	1	553 (488–810)	636 (507–850)	1012 (736–1293)	0.1
	2	526 (448–719)	603 (483–886)	1955 (1033–3058)	0.037 ^a^
Total calcium, mmol/L	1	2.11 (1.90–2.37)	2.13 (2.04–2.24)	1.95 (1.87–2.27)	0.5
	2	2.05 (2.00–2.16)	2.11 (2.01–2.19)	1.90 (1.45–2.02)	0.020 ^b^
Urea, mmol/L	1	3.67 (2.83–6.00)	4.67 (3.50–6.00)	6.67 (5.00–13.0)	0.020 ^a^
	2	3.17 (2.83–4.00)	4.67 (3.17–6.17)	15.0 (8.17–18.50)	<0.001 ^a,b,c^
Creatinine, µmol/L	1	65.4 (59.2–80.4)	69.8 (60.1–87.5)	92.4 (75.6–171.0)	0.036 ^a^
	2	61.0 (46.9–68.1)	60.1 (51.3–71.6)	123.3 (68.5–204.6)	0.014 ^a,b^
KIM-1, ng/mL	1	2.73 (1.30–5.11)	3.41 (2.07–6.14)	2.59 (1.63–4.74)	0.6
	2	2.15 (1.31–3.96)	2.75 (1.66–5.53)	1.82 (1.58–4.01)	0.2
L-FABP, ng/mL	1	5.82 (4.59–13.97)	22.52 (8.18–53.06)	12.84 (6.78–18.90)	0.2
	2	4.51 (3.84–7.56)	17.36 (4.01–30.34)	10.18 (8.03–12.33)	0.3

Ang-2—angiopoietin 2; CRP—C-reactive protein; KIM-1—kidney injury molecule-1; LDH—lactate dehydrogenase; L-FABP—liver-type fatty acid binding protein; MAP—mild acute pancreatitis; MSAP—moderately severe acute pancreatitis; NEU—neutrophils; PCT—procalcitonin; SAP—severe acute pancreatitis; sFlt-1—soluble fms-like tyrosine kinase -1; WBC—white blood cells; ^a^ significant difference between MAP and SAP groups in post-hoc comparison; ^b^ significant difference between MSAP and SAP groups in post-hoc comparison; ^c^ significant difference between MAP and MSAP groups in post-hoc comparison.

**Table 3:** Correlations between IL-6 and selected laboratory markers in patients with AP during first 48 h of disease.

Variables	Study day 1		Study day 2	
	R	*p*	R	*p*
sFlt-1	0.38	0.001	0.24	0.1
Ang-2	0.26	0.1	0.54	0.004
Total calcium	−0.34	0.003	−0.44	<0.001
Total protein	−0.48	0.001	−0.48	<0.001
Albumin	−0.34	0.005	−0.57	<0.001
CRP	0.35	0.001	0.67	<0.001
PCT	0.44	<0.001	0.70	<0.001
WBC	0.37	<0.001	0.50	<0.001
NEU	0.42	0.006	0.63	<0.001
Urea	0.10	0.4	0.22	0.044
KIM-1	0.50	<0.001	0.25	0.045
L-FABP	0.14	0.4	0.51	<0.001
LDH	0.27	0.020	0.63	<0.001
Bilirubin	0.09	0.4	0.30	0.005

Ang-2—angiopoietin 2; AP—acute pancreatitis; CRP—C-reactive protein; HCT—hematocrit; IL-6—interleukin 6; LDH—lactate dehydrogenase; NEU—neutrophils; PCT—procalcitonin; sFlt-1—soluble fms-like tyrosine kinase-1; WBC—white blood cells.

**Table 4:** Simple logistic regression models to predict severe course of AP based on serum IL-6 concentrations.

Dependent Variable	Odds Ratio (95% Confidence Interval per 100 pg/mL); *p*-Value	
	IL-6 on Admission	IL-6 on Day 2
SAP (2012 Atlanta)	1.17 (1.03-1.32); *p* = 0.011	1.18 (1.01-1.37); *p* = 0.030
Cardiovascular, lung or kidney failure	1.27 (1.09-1.48); *p* = 0.002	1.19 (1.02-1.39); *p* = 0.023
ICU transfer or death	1.22 (1.06-1.41); *p* = 0.004	1.15 (1.00-1.34); *p* = 0.051
Death	1.23 (0.06-1.43); *p* = 0.007	1.02 (0.76-1.36); *p* = 0.9
